# Effects of Thermal Conductive Materials on the Freeze-Thaw Resistance of Concrete

**DOI:** 10.3390/ma14154063

**Published:** 2021-07-21

**Authors:** Byeong-Hun Woo, Dong-Ho Yoo, Seong-Soo Kim, Jeong-Bae Lee, Jae-Suk Ryou, Hong-Gi Kim

**Affiliations:** 1 Civil and Environmental Engineering Department, Hanyang University, Jaesung Civil Engineering Building, 222 Wangsimni-ro, Seongdong-Gu, Seoul 04763, Korea; dimon123@hanyang.ac.kr (B.-H.W.); dongho3461@naver.com (D.-H.Y.); jsryou@hanyang.ac.kr (J.-S.R.); 2 Department of Civil Engineering, Daejin University, 1007 Hoguk-ro, Pocheon-si 11159, Korea; sskim@daejin.ac.kr; 3 GFC R&D Co., Ltd., 155 Hoguk-ro, Pocheon-si 11158, Korea; dlwjdqo@nate.com

**Keywords:** freeze–thaw, silicon carbide, graphite, steel fiber, thermal conductive material

## Abstract

To solve the problem of black ice, many studies are being carried out. The key in recent days is enhancing the thermal conductivity of concrete. In this study, to improve the thermal conductivity, silicon carbide was used to substitute 50% and 100% of the fine aggregate. In addition, steel fiber is not only for enhancing the mechanical properties but could enhance thermal conductive material. Hence, the arched-type steel fiber was used up to a 1% volume fraction in this study. Furthermore, graphite was used for 5% of the volume fraction for enhancing the thermal conductivity. However, thermal damage would occur due to the difference in thermal conductivity between materials. Therefore, the thermal durability must be verified first. The target application of the concrete in this study was its use as road paving material. To evaluate the thermal durability, freeze–thaw and rapid cyclic thermal attacks were performed. The thermal conductivity of the specimens was increased with the increase in thermal conductive materials. Graphite has already been reported to have a negative effect on mechanical properties, and the results showed that this was the case. However, the steel fiber compensated for the negative effect of graphite, and the silicon carbide provided a filler effect. Graphite also had a negative effect on the freeze–thaw and rapid cyclic thermal attack, but the steel fiber compensated for the reduction in thermal durability. The silicon carbide also helped to improve the thermal durability in the same way as steel fiber. Comprehensively, the steel fiber enhanced all of the properties of the tests. Using 100% silicon carbide was considered the acceptable range, but 50% of silicon carbide was the best. Graphite decreased all the properties except for the thermal conductivity. Therefore, the content of graphite or using other conductive materials used should be carefully considered in further studies.

## 1. Introduction

Cold regions have two kinds of threatening factors for vehicle users. One is black ice and the other is pot-holes caused by the freeze–thaw cycle. Black ice makes the surface of the road slippery and causes traffic accidents [1]. To prevent the generation of black ice, people use chemical salts such as CaCl_2_ [2,3,4]. However, it was demonstrated that chemical salts bring about the deterioration of concrete and reduce the service life of the concrete [2]. In particular, Matalkah et al. [4] showed that the combination of freeze–thaw conditions and chemical salts accelerated the deterioration of concrete. Thus, researchers devised heating systems for reducing the use of chemical salts and preventing the formation of black ice [5,6,7]. The studied pavement heating systems showed a good performance on snow-melting; however, the big problem was that reaching time to the surface of pavement took a long time [5,6,7]. To overcome this problem, research was conducted to enhance the thermal conductivity of the materials themselves. Representative cases include the application of Carbon Nanofibers (CNFs) and Carbon Nanotubes (CNTs) [8,9,10,11,12,13]. Although CNFs/CNTs have a superior thermal conductivity of 3000 to 6000 W/mK [11], there were critical limits to improve the thermal conductivity of the cement composites. Because CNFs/CNTs are a kind of fiber, therefore the CNFs/CNTs could not apply up to 2% of the volume fraction [8,9,10,11,12,13]. In addition, CNFs/CNTs should separate each element using physical or chemical methods because of the Van der Waals force [8].

Graphite has good thermal conductivity because graphite is a kind of carbon material and has been extensively investigated by researchers [14,15,16]. However, it is reported that the mechanical properties are significantly decreased by using graphite above 5% of volume fraction [17,18]. In addition, the other problem of using CNFs/CNTs and graphite is the cost. To overcome the limits of previous studies, the substitution method was applied to this study because the aggregate occupies more than 65% of the volume fraction [19]. Silicon carbide (SiC) was chosen as the substituting material of the fine aggregate; as SiC has good thermal conductivity and hardness, it is considered sufficient as a fine aggregate substitution material [20,21,22].

In this study, SiC was substituted for 50% and 100% of fine aggregate in order to improve the thermal conductivity. In addition, graphite was used at 5% of volume for enhancing the thermal conductivity, and the arched-type steel fiber was used for compensating the reduction in mechanical properties by the graphite [23,24,25,26,27]. Furthermore, steel fiber could be used as the thermal conductive material because the steel fiber has a high level of thermal conductivity [28]. Due to applying the various thermal conductive materials to the concrete, thermal damage would be generated by the difference in the thermal conductivity of each material in the cold environment, e.g., via freeze–thaw [29].

The concrete introduced in this study was intended for use as road pavement, which meant it was necessary for the thermal durability performance in conditions such as freeze–thaw to be verified. Therefore, the purpose of this study is to assess the thermal durability of the concrete with thermal conductive materials. To assess the thermal durability, two main experiments were performed: freeze–thaw (FT) and rapid cyclic thermal attack (RCTA). The concrete used in this study was made for application as road paving material, therefore, the FT resistance was important. In addition, cold regions usually change the air temperature very rapidly. Therefore, the RCTA test was essential for assessing the thermal durability of concrete.

## 2. Materials and Experiments

### 2.1. Materials

**Figure 1:**
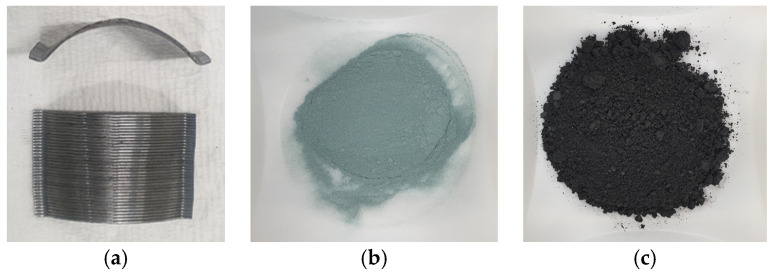
Used thermal conductive materials. (**a**) Arched-type steel fiber; (**b**) SiC; (**c**) Graphite.

In this study, Ordinary Portland Cement (OPC) [30], graphite, SiC, and steel fiber were mainly used (Figure 1). The chemical composition of OPC is indicated in Table 1. The properties of graphite are indicated in Table 2 and the properties of SiC are summarized in Table 3. Steel fiber that has an arched shape was used and the details are in Table 4.

**Figure 2:**
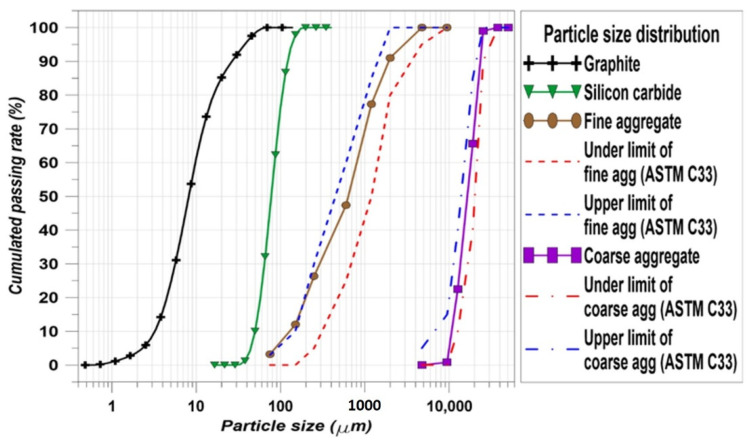
PSDs of the graphite, SiC, fine aggregate, and coarse aggregate.

The fine and coarse aggregates used natural sand and crushed stone, respectively. The specific gravity of the fine aggregate was 2.62 ton/m^3^ and the coarse aggregate was 2.71 ton/m^3^. Except for the OPC, graphite, SiC, the fine aggregate, and the coarse aggregate have their own particle size distribution (PSD). The PSD properties of each material are important to investigate how the concrete is made well. The PSDs of graphite, SiC, the fine aggregate, and the coarse aggregate are indicated in Figure 2. In particular, there is a standard for fine and coarse aggregates [31]. ASTM C33 [31] recommends the PSD upper and lower limits for the aggregates. According to the PSDs of the fine and coarse aggregates in this study, the particle distributions of the aggregates are located between the upper and lower limits. Therefore, it can be considered that the conditions of the aggregates are acceptable.

**Figure 3:**
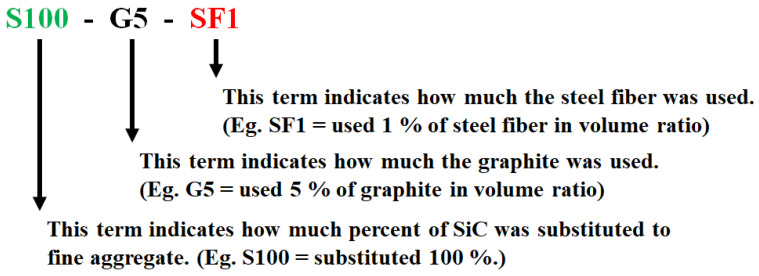
Specimen naming.

A total 6 cases of concrete specimens were cast. The specimens were cured for one day in air and demolded after one day of curing. After demolding, the specimens were immersed in the water at 25 ℃ for 27 days (total 28 days curing). The mixing properties of the specimens are summarized in Table 5 and the naming details of the specimens are indicated in Figure 3.

### 2.2. Experiments

**Figure 4:**
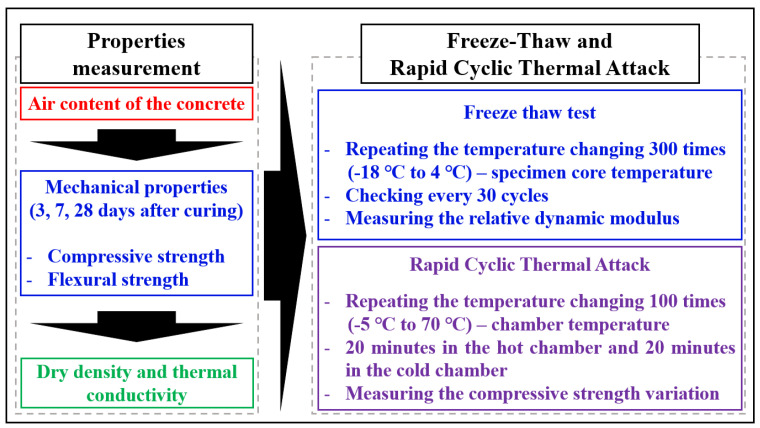
The details of the research flow.

The main experiments were the FT cycle test and RCTA and the supporting experiment was strength test and thermal properties. The detail of the research flow is indicated in Figure 4.

According to Figure 4, the air content of the specimens is the fresh property of concrete, and this is important to the FT test. There is a wide range of air content and the range of 3.5 to 4.5% is commonly used [32]. The target air content of this study was 4% in each specimen, and the results of the air content are indicated in Table 6. The acceptable range of the air content was 4% ± 0.5%.

#### 2.2.1. Compressive Strength Test

The compressive strength was measured at 3, 7, and 28 days of curing. In addition, the stress–strain curve was measured at 28 days of curing in each specimen. Test procedure followed ASTM standard [33]. When measuring the stress–strain curve, a universal testing machine (UTM) which has 200 tons of load capacity (Shimadzu, CCM-200A, Shimadzu Corporation, Japan) was used for measuring the compressive strength. In addition, the strain sensor, which has a 0.6% sensitivity, was used for measuring the concrete strain (Tokyo Measuring Instruments Lab (TML), PL-60-11-3LJC-F: One gauge—three wires, Tokyo Measuring Instruments Laboratory Corporation, Tokyo, Japan). From the test method, the static elastic modulus could be measured by using the result data. The calculation of elastic modulus followed Equation (1) [34].

(1) $$E=\frac{S_{0.4}-S_{init}}{\varepsilon_{0.4}-\varepsilon_{init}}$$

where, *E* is the elastic modulus (MPa), *S*_0.4_ is the stress corresponding to 40% of ultimate stress (MPa), $S_{init}$ is the stress corresponding to a longitudinal strain of $\varepsilon_{init}$ of 0.00005 (MPa), $\varepsilon_{0.4}$ is the longitudinal strain produced by the *S*_0.4_, and $\varepsilon_{init}$ is the strain value of 0.00005.

#### 2.2.2. Flexural Strength Test

**Figure 5:**
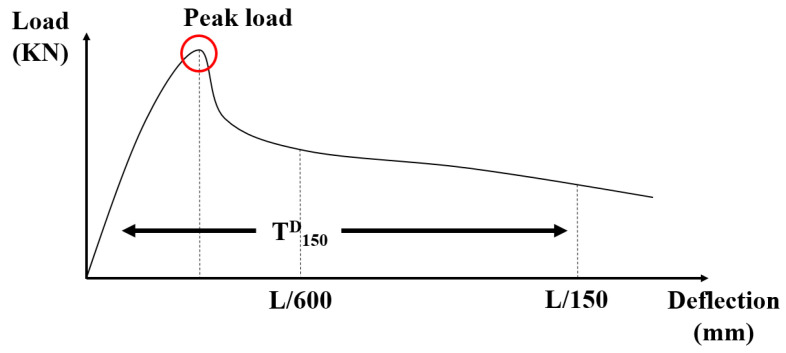
The flexural behavior of the steel fiber reinforced concrete.

The flexural strength was measured at the same curing ages as the compressive strength. The load–deflection curve was measured at 28 days of curing in each specimen and followed the test method of ASTM [35]. In this standard [35], there are two types of strength calculations: peak flexural strength and equivalent flexural strength. Due to the fiber reinforcing to the concrete, the behavior after cracking should be considered. Therefore, the equivalent flexural strength should be calculated. The flexural strength was calculated by Equation (2) [35].

(2) $$f_{r}=\frac{PL}{bd^{2}}$$

where, $f_{r}$ is the flexural strength (MPa), P is the load (N), L is the length of span (mm), b is the width of the specimen section (mm), and d is the height of the specimen section (mm). According to Figure 5, The deflection points of L/600 and L/150 are important to calculate the equivalent flexural strength.

The flexural test was conducted in the case of the 4-point bending test and the spans (L) were 300 mm in this experiment. Therefore, the checking points of net deflection in Figure 5 were 0.5 mm and 2 mm. The calculation of equivalent flexural strength was followed Equation (3) [35].

(3) $$f_{eq}=\frac{150\times T_{150}^{D}}{bd^{2}}$$

where, $f_{eq}$ is the equivalent flexural strength (MPa) and $T_{150}^{D}$ is the area of the load–deflection curve from 0 to L/150 (N × mm). The same UTM as the compressive strength was used to measure the flexural strength.

#### 2.2.3. Dry Density

Thermal conductive materials were used in this study. Therefore, the thermal conductivity was measured. Before considering the thermal conductivity of the specimens, the dry density has to be considered because the thermal conductivity is sensitive to the density of materials. There is a simple standard for measuring the dry density and water absorption following standard [36]. The size of the specimen was 100 mm in diameter and 50 mm in height. Specimens were immersed in the de-ionized water for 3 days and dried for 2 days at 80 °C in a dry chamber.

#### 2.2.4. Thermal Conductivity

It is important to measure the thermal conductivity because thermal conductive materials were used in this study. There are many methods used to measure the thermal conductivity of cement composites [37]. However, we chose a simple method involving the use of a machine: Isomet-2114 (Applied Precision, Bratislava, Slovakia) [38,39,40]. Isomet-2114 is widely used to measure the thermal conductivity of the concrete specimens and the results are accurate. When measuring the thermal conductivity, the cylindrical specimens were used that had a size of 100 mm in diameter and 50 mm in height.

#### 2.2.5. Freeze–Thaw Test

**Figure 6:**
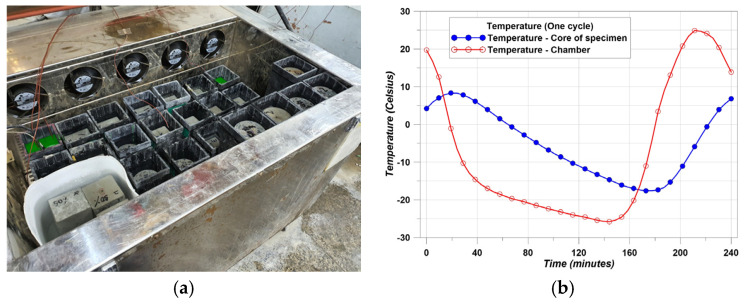
Testing chamber and temperature cycle. (**a**) Freeze-thaw chamber; (**b**) One cycle of temperature.

The concrete made with thermal conductive materials was intended for use as concrete pavement on the road. Therefore, freeze–thaw resistance is an important factor for road users. Figure 6a shows the freeze–thaw chamber and Figure 6b shows the cycle of temperature. Figure 6b shows the real test data.

The temperature cycle has to meet the condition that the core temperature of the specimens should repeat −18 ± 2 ℃ to 6 ± 2 ℃ for 4 h in one cycle. The cycle was repeated a total of 300 times and the Relative Dynamic Modulus (RDM) was measured every 30 cycles. In addition, mass loss was measured every 30 cycles. The test method followed the ASTM standard [41] and followed Equation (4) in order to calculate RDM.

(4) $$P_{r}=\frac{n_{m}^{2}}{n_{0}^{2}}\times 100$$

where, $P_{r}$ is RDM (%), $n_{m}$ is the measured frequency after m cycles of FT, and $n_{0}$ is the measured frequency at 0 cycles of FT.

#### 2.2.6. Rapid Cyclic Thermal Attack Test

**Figure 7:**
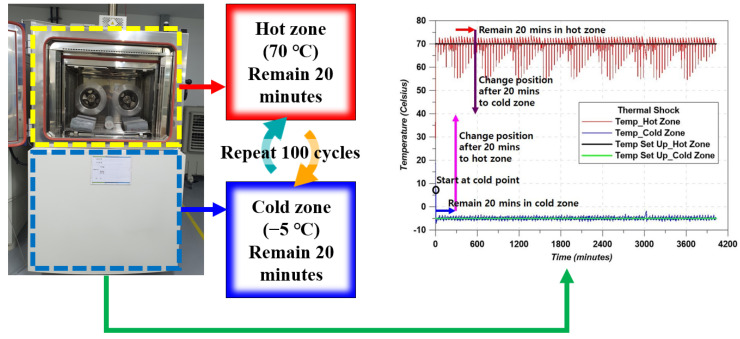
RCTA test concept.

Due to the difference in the thermal conductivity of each material, it is impossible to neglect the effect of thermal damage. This test is similar to the FT test, but the applied conditions and the concept were different. The purpose of this test is to show how well the specimens resist harsh thermal conditions. The test concept is indicated in Figure 7. The mechanical variation was evaluated by performing the compressive test with the stress–strain curve.

## 3. Results and Discussion

### 3.1. Compressive Strength Restuls

**Figure 8:**
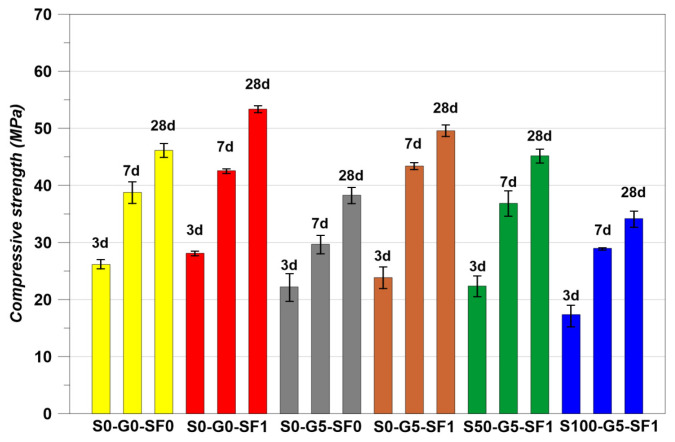
Compressive strength results.

**Figure 9:**
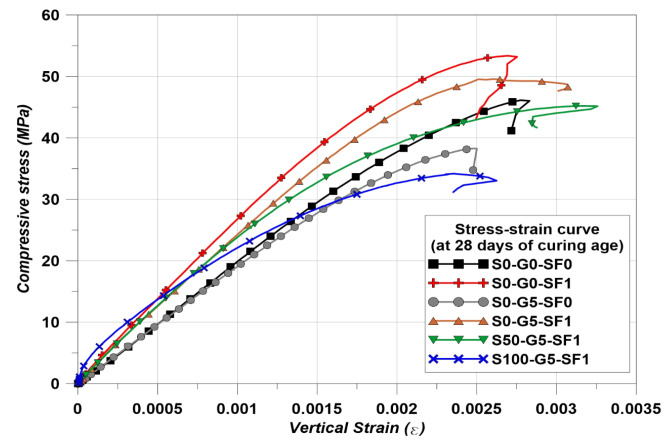
Compressive stress–strain curves.

The compressive strength results are indicated in Figure 8 and the stress–strain curves of the specimens at the 28 days of curing are indicated in Figure 9. The S0-G0-SF1 showed the best compressive performance. The cases using steel fiber in concrete have been reproduced many times and have reported that using steel fiber brings an increase in compressive strength [42,43,44]. The S0-G5-SF0 showed less strength than S0-G0-SF0. Many studies showed that graphite has a negative effect on the mechanical strength of concrete [14,15,16,17,18,28]. In addition, a significant decrease in mechanical properties could be found when the graphite used more than 10% of the volume fraction [14,15,16,17,18,28]. Although the mechanical properties are decreased when using graphite, there is an advantage for the thermal conductivity. Therefore, the 5% of volume fraction was chosen for use in the graphite used in this study [14,15,16,17,18,28]. The S0-G5-SF1 specimen showed higher compressive strength than S0-G0-SF0. However, S0-G5-SF1 showed less strength than S0-G0-SF1. This is the same reason for the studies of graphite [14,15,16,17,18,28]. In addition, using SiC as a fine aggregate up to 50% showed the almost same compressive performance compared to the normal cement composites [45]. However, the S50-G5-SF1 showed a slightly smaller value than S0-G0-SF0 and this was considered a result of graphite used. The S100-G5-SF1 showed the lowest value. A study showed that the mechanical properties decreased when using 100% SiC as fine aggregate [45].

Table 7 is indicating the measured elastic modulus of the specimens. The S0-G5-SF0 showed the lowest elastic modulus value. However, the S100-G5-SF1 showed the lowest compressive strength. The other specimens showed that the elastic modulus was increased with the increase in compressive strength. However, the S0-G5-SF0 and S100-G5-SF1 specimens showed a different trend in the results of elastic modulus. This phenomenon could be explained in Figure 9. S0-G5-SF0 did not use steel fiber. Therefore, this specimen could be treated as a brittle material. Hence, the initial strain behavior was almost the same as S0-G0-SF0. However, the specimens which used the steel fiber showed more steep initial strain behavior than S0-G0-SF0 and S0-G5-SF0. In this regard, the elastic modulus results of S0-G5-SF0 and S100-G5-SF1 could explain why the elastic modulus of S100-G5-SF1 appeared to have a larger value than S0-G5-S0.

### 3.2. Flexural Strength Restuls

**Figure 10:**
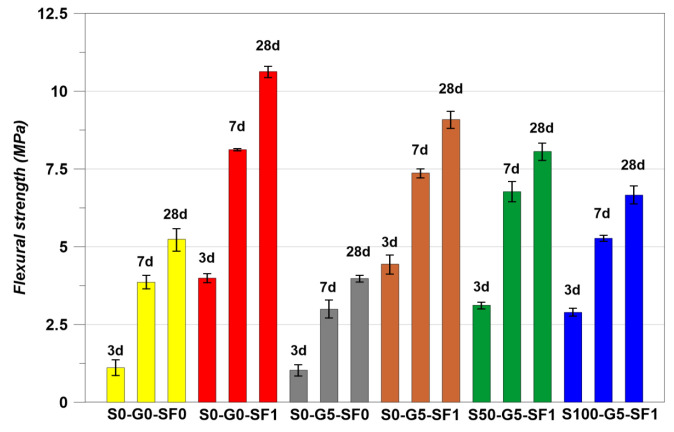
Flexural strength results.

**Figure 11:**
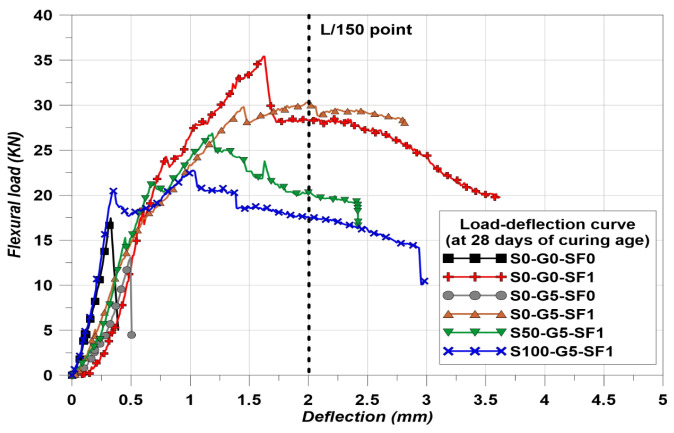
Flexural load–deflection curves.

The flexural strength results are indicated in Figure 10 and the load–deflection curves of the specimens at 28 days of curing are indicated in Figure 11. The trend of flexural strength is similar to the compressive strength results, except for S0-G5-SF0 and S100-G5-SF1. In the case of the compressive strength, S100-G5-SF1 showed the lowest value. However, the lowest flexural strength was found for S0-G5-SF0. This is considered to have happened as a result of the inclusion of steel fiber. In addition, the arched type of steel fiber was used in this study; the anchorage effect of the arched type steel fiber helped to improve the flexural strength [46].

The $f_{eq}$ for each specimen was calculated and the results are indicated in Table 8. The S0-G0-SF0 and S0-G5-SF0 could not measure the $f_{eq}$ because these specimens were not used the steel fiber (brittle condition). The $f_{eq}$ is based on the area of the load–deflection curve, the results of Table 8 demonstrate well the flexural performance after cracking.

### 3.3. Dry Density Results

Thermal conductivity is sensitive to the porosity of materials [47] and the porosity is related to the water absorption [48]. It can be considered that there are many voids when the water absorption rate is large [47,48]. Table 9 indicates the water absorption and dry density. The results of three specimens were averaged in each case.

S0-G5-SF1 showed the highest value of water absorption. This is because using fibers in concrete makes microcracks between cement matrix and the fibers [49]. In addition, graphite is also making concrete porous [15]. The combination of these phenomena, causing the water absorption of S0-G5-SF1, was measured as in in Table 9. Comparing the results of S0-G0-SF1, S0-G5-SF0, and S0-G5-SF1, this trend was reflected in the results of the thermal conductivity.

### 3.4. Thermal Conductivity Results

**Figure 12:**
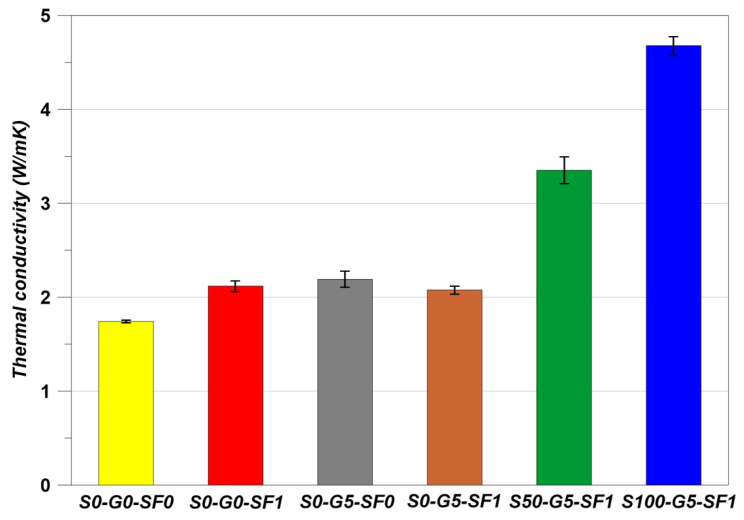
Thermal conductivity.

The thermal conductivity results are indicated in Figure 12. The S0-G5-SF0 showed a higher thermal conductivity than S0-G0-SF0 despite the negative effects of the graphite on the mechanical properties. As the graphite has high thermal conductivity, the thermal conductivity of S0-G5-SF0 appeared as in Figure 12 [15,16,17]. Steel fiber has also high thermal conductivity, so the S0-G0-SF1 showed almost the same thermal conductivity results as the S0-G5-SF0 specimens [28]. However, S0-G5-SF1 showed a slight decrease in thermal conductivity and this phenomenon is related to the water absorption rate in discussed in Section 3.3. The water absorption rate showed the highest value when using the steel fiber and graphite simultaneously. Therefore, the thermal conductivity of S0-G5-SF1 showed a slight decreasing behavior.

In S50-G5-SF1 and S100-G5-SF1, the thermal conductivity was increased to a large gap in comparison to other specimens. In the aspect of concrete volume, the fine aggregate occupies 30% of concrete volume [19]. S50-G5-SF1 was substituted in the fine aggregate with 50% of SiC (occupying 15% of concrete volume) and S100-G5-SF1 was substituted with the fine aggregate up to 100% of SiC (occupying 30% of concrete volume). Therefore, the thermal conductivity was affected by SiC much more than the S0-G0-SF1, S0-G5-SF0, and S0-G5-SF1 specimens [45].

### 3.5. Freeze–Thaw Test Results

**Figure 13:**
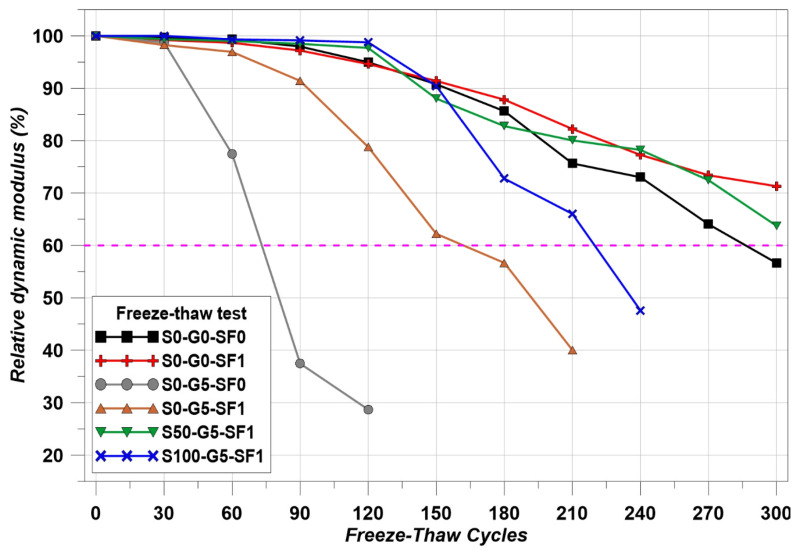
Freeze–thaw test results.

**Figure 14:**
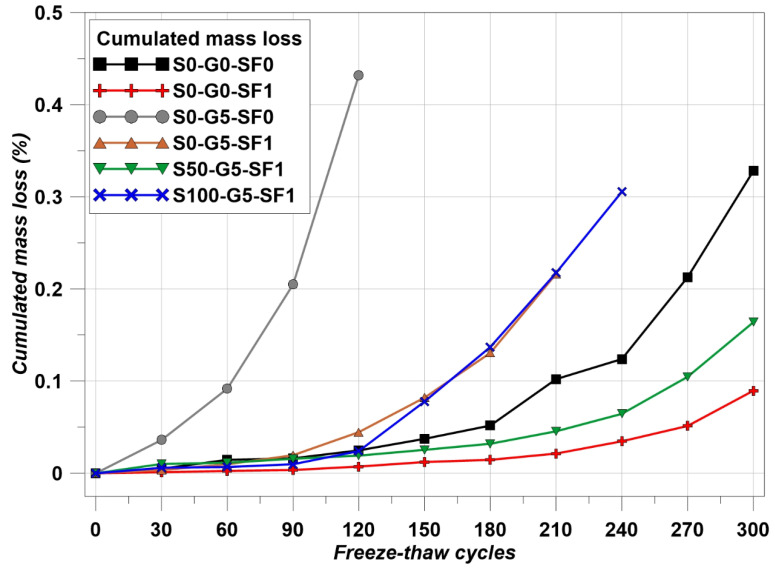
Mass loss results.

The results of RDM are indicated in Figure 13 and the mass loss results are indicated in Figure 14. S0-G5-SF0 was terminated in the FT test at 120 cycles. The mass loss of S0-G5-SF0 was accelerated after 30 cycles of the FT test. Due to the poor mechanical properties of using graphite, the FT test of S0-G5-SF0 was terminated at an early state [50]. On the other hand, the specimens which used the steel fiber showed an improvement of FT resistance. Compared to S0-G5-SF0 and S0-G5-SF1, S0-G5-SF1 maintained an RDM of above 60% for 150 cycles, and the mass loss was decreased. In addition, compared to S0-G0-SF0 and S0-G0-SF1, S0-G0-SF1 showed a marginal ability to resist the FT cycles. However, the S0-G0-SF0 showed the RDM as being under 60% at 300 cycles. These results show that the steel fiber improved mechanical performance and thermal conductivity and improved the FT resistance [25,51].

Using SiC has many benefits for concrete such as improving the durability of the cement composites via a filler effect and improving the thermal attack resistance. Although using 100% of SiC as a fine aggregate led to a decrease in mechanical properties [45], the benefits of using SiC could be confirmed when using the SiC up to 50% [45]. Comparing S0-G5-SF1 and S50-G5-SF1, the FT resistance of S50-G5-SF1 was dramatically improved. S50-G5-SF1 showed above 60% of RDM at the last cycle and the mass loss was reduced compared to S0-G5-SF1. S100-G5-SF1 showed relatively poorer FT resistance than S50-G5-SF1. Fully substituting the fine aggregate for SiC may help to improve the thermal conductivity; however, there were many disadvantages to other performances such as compressive and flexural strength. Usually, monotonous PSD brings about a reduction in the concrete performance and SiC has monotonous PSD (see Figure 2) [45]. Therefore, 50% of SiC and steel fiber compensated the negative effect from the graphite and the combination of SiC and steel fiber improved the FT resistance significantly.

### 3.6. Rapid Cyclic Thermal Attack Results

The effect of RCTA was observed by comparing the compressive strength. The results are indicated in Table 10. The trend of RCTA results appeared similar to the FT test. However, the range of the temperature cycle was much harsher than the FT test. Therefore, the degradation of the compressive strength appeared remarkable. S0-G5-SF0 showed the largest reduction ratio. Comprehensively, the specimen case using only graphite showed the decreasing the performance of concrete except for thermal conductivity. S0-G0-SF1 showed the lowest reduction ratio. S0-G0-SF1 showed the best performance in this study. Using arched type steel fiber brings many benefits for the performance of the concrete. Arched type steel fiber could maximize the anchorage effect compared to other steel fibers because the arched type steel fiber anchored the whole body into the concrete matrix [46,52,53]. Therefore, it is considered that the RCTA result of S0-G0-SF1 was affected by this anchorage effect [46,52,53]. In addition, the compensation of the reduction ratio could be confirmed through S0-G5-SF1. The reduction ratio of S0-G5-SF0 was 44.96% and S0-G5-SF1 was 30.17%. Therefore, 14.79% of the reduction ratio was compensated.

SiC also improves the thermal attack resistance [29]. S50-G5-SF1 and S100-G5-SF1 showed that the reduction ratio by RCTA was decreased by 7.38% and 1.36%, respectively, compared to S0-G5-SF1. In particular, although S100-G5-SF1 showed poor mechanical properties, RCTA resisting performance was increased by using SiC compared to S0-G0-SF0 and S0-G5-SF1 [29].

## 4. Conclusions

This study focused on the effect of thermal conductive materials on the FT resistance of concrete. The effect of thermal conductive materials on concrete was confirmed by various experiments. The comprehensive conclusions of this study are as follows:

1. Arched type steel fiber improves the mechanical properties of concrete due to the anchorage effect. On the contrary, it was demonstrated that using graphite brought about a negative effect on the mechanical properties. However, graphite is a good material for improving the thermal conductivity of concrete. Therefore, the decrease in mechanical properties caused by using graphite could be compensated by using arched type steel fiber.
2. SiC is able to be used as fine aggregate and has sufficient thermal conductivity. In addition, it was demonstrated that the steel fiber could be used as a thermal conductive material through the thermal conductivity results. The combination of SiC and steel fiber maximized the improvement in the thermal conductivity of concrete. Adding graphite also brought about an increase in thermal conductivity.
3. It was demonstrated that using graphite is not suitable for FT and RCTA resistance through the results of the FT test and RCTA test. However, the arched type steel fiber showed a remarkable improvement of the FT resistance and RCTA. In addition, SiC compensated for the negative effect of graphite on the FT and RCTA.

## Figures and Tables

**Table 1:** Properties of OPC.

**Chemical Composition (%)**					
**SiO_2_**	**Al_2_O_3_**	**Fe_2_O_3_**	**CaO**	**MgO**	**Other**
20.8	6.3	3.2	62	3.3	4.4
**Physical properties**					
**Blaine (cm^2^/g)**			**Specific gravity (ton/m^3^)**		
3200			3.15		

**Table 2:** Properties of graphite.

Chemical Composition (%)		Other Properties	
C	Other	Specific Gravity (ton/m^3^)	Thermal Conductivity (W/mK)
86%	14%	0.83	110

**Table 3:** Properties of SiC.

**Chemical Composition (%)**			
**SiC**	**Fe_2_O_3_**	**Fe_3_C**	**Other**
94	0.7	0.5	4.8
**Other properties**			
**Tensile strength (MPa)**	**Elastic modulus** **(GPa)**	**Specific gravity (ton/m^3^)**	**Thermal conductivity (W/mK)**
620	192	3.22	25.5–40

**Table 4:** Properties of steel fiber.

Tensile Strength (MPa)	Specific Gravity (ton/m^3^)	Aspect Ratio (%)	Thermal Conductivity (W/mK)
1500	7.85	56 (Length: 42 mm, Diameter: 0.75 mm)	57

**Table 5:** Mixing properties of the specimens (unit: kg/m^3^).

Specimen	W/C (%)	Water	Cement	Fine Agg	Coarse Agg	Water Reducer	SiC	Graphite	Steel Fiber
S0-G0-SF0	55	225.5	410	764	1032	4.1	0	8.26	0
S0-G0-SF1				764			0		78.5
S0-G5-SF0				764			0		0
S0-G5-SF1				764			0		78.5
S50-G5-SF1				382			382		78.5
S100-G5-SF1				0			764		78.5

**Table 6:** Air content results.

Specimen	S0-G0-SF0	S0-G0-SF1	S0-G5-SF0	S0-G5-SF1	S50-G5-SF1	S100-G5-SF1
Air content (%)	4.2	4.4	3.6	4.0	3.8	4.2

**Table 7:** Results of the elastic modulus.

Specimen	S0-G0-SF0	S0-G0-SF1	S0-G5-SF0	S0-G5-SF1	S50-G5-SF1	S100-G5-SF1
Elastic modulus (MPa)	24,561	26,860	21,981	24,432	24,111	22,663

**Table 8:** Results of the equivalent flexural strength.

Specimen	S0-G0-SF0	S0-G0-SF1	S0-G5-SF0	S0-G5-SF1	S50-G5-SF1	S100-G5-SF1
$f_{eq}$	Could not measure	6.465	Could not measure	6.252	5.538	5.316

**Table 9:** Results of the dry density and water absorption.

Specimen	S0-G0-SF0	S0-G0-SF1	S0-G5-SF0	S0-G5-SF1	S50-G5-SF1	S100-G5-SF1
Water absorption (%)	4.682	4.923	5.593	7.076	5.694	6.449
Dry density (kg/m^3^)	2098.24	2145.61	1967.79	2019.11	2110.11	2047.97

**Table 10:** Results of RCTA.

Specimen	S0-G0-SF0	S0-G0-SF1	S0-G5-SF0	S0-G5-SF1	S50-G5-SF1	S100-G5-SF1
Before RCTA (MPa)	46.14	53.37	38.28	49.58	45.19	34.19
After RCTA (MPa)	30.82	45.81	21.07	34.62	34.89	24.34
Reduction ratio (%)	33.20	14.16	44.96	30.17	22.79	28.81

## Data Availability

Not applicable.
